# Evidence for hypoxia‐induced dysregulated cholesterol homeostasis in preeclampsia: Insights into the mechanisms from human placental cells and tissues

**DOI:** 10.1096/fj.202301708RR

**Published:** 2024-01-24

**Authors:** Barbara Fuenzalida, Maria Jose Yañez, Martin Mueller, Hiten D. Mistry, Andrea Leiva, Christiane Albrecht

**Affiliations:** ^1^ Institute of Biochemistry and Molecular Medicine, Faculty of Medicine University of Bern Bern Switzerland; ^2^ School of Medical Technology, Faculty of Medicine and Science Universidad San Sebastián Santiago Chile; ^3^ Division of Gynecology and Obstetrics Lindenhofgruppe Bern Switzerland; ^4^ Department for BioMedical Research University of Bern Bern Switzerland; ^5^ Department of Women and Children's Health School of Life Course and Population Health Sciences, King's College London London UK; ^6^ Swiss National Center of Competence in Research, NCCR TransCure University of Bern Bern Switzerland

**Keywords:** cholesterol, hypoxia, placenta, preeclampsia, reactive oxygen species, trophoblast

## Abstract

Preeclampsia (PE) poses a considerable risk to the long‐term cardiovascular health of both mothers and their offspring due to a hypoxic environment in the placenta leading to reduced fetal oxygen supply. Cholesterol is vital for fetal development by influencing placental function. Recent findings suggest an association between hypoxia, disturbed cholesterol homeostasis, and PE. This study investigates the influence of hypoxia on placental cholesterol homeostasis. Using primary human trophoblast cells and placentae from women with PE, various aspects of cholesterol homeostasis were examined under hypoxic and hypoxia/reoxygenation (H/R) conditions. Under hypoxia and H/R, intracellular total and non‐esterified cholesterol levels were significantly increased. This coincided with an upregulation of HMG‐CoA‐reductase and HMG‐CoA‐synthase (key genes regulating cholesterol biosynthesis), and a decrease in acetyl‐CoA‐acetyltransferase‐1 (ACAT1), which mediates cholesterol esterification. Hypoxia and H/R also increased the intracellular levels of reactive oxygen species and elevated the expression of hypoxia‐inducible factor (HIF)‐2α and sterol‐regulatory‐element‐binding‐protein (SREBP) transcription factors. Additionally, exposure of trophoblasts to hypoxia and H/R resulted in enhanced cholesterol efflux to maternal and fetal serum. This was accompanied by an increased expression of proteins involved in cholesterol transport such as the scavenger receptor class B type I (SR‐BI) and the ATP‐binding cassette transporter G1 (ABCG1). Despite these metabolic alterations, mitogen‐activated‐protein‐kinase (MAPK) signaling, a key regulator of cholesterol homeostasis, was largely unaffected. Our findings indicate dysregulation of cholesterol homeostasis at multiple metabolic points in both the trophoblast hypoxia model and placentae from women with PE. The increased cholesterol efflux and intracellular accumulation of non‐esterified cholesterol may have critical implications for both the mother and the fetus during pregnancy, potentially contributing to an elevated cardiovascular risk later in life.

## Graphical abstract

This study provides a comprehensive overview of disrupted cholesterol homeostasis pathways in the pregnancy disorder preeclampsia. Additionally, we investigated a cell model designed to simulate this disorder by inducing continuous and intermittent hypoxic episodes. The graphical abstract illustrates the examined pathways, which include cholesterol biosynthesis, conversion to progesterone, intracellular storage via esterification, and cholesterol transport. Moreover, the consequences of prolonged exposure to hypoxia on the generation of reactive oxygen species and gene regulatory processes are outlined.

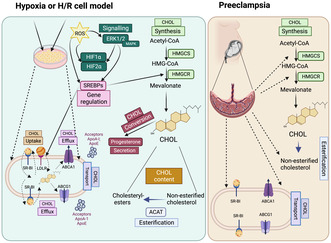

**Abbreviations**
ABCA1: ATP‐binding cassette transporter A1
ABCG1: ATP‐binding cassette transporter G1
ACAT1: acetyl‐CoA acyltransferase 1
ApoA‐I: apolipoprotein A type I
ApoE: apolipoprotein E
BSA‐FFA: bovine serum albumin‐free fatty acids
CVD: cardiovascular disease
CK7: cytokeratin 7
DCFH2‐DA: 2′,7′‐dichlorodihydrofluorescein diacetate
ERK1/2: extracellular‐signal‐regulated kinase
DAPI: 4',6‐Diamidino‐2‐Phenylindole
GAPDH: Glyceraldehyde 3‐phosphate dehydrogenase
GST: glutathione‐S‐transferase
HDL: high density lipoprotein
HIF: hypoxia‐inducible factor
HMGCR: 3‐hydroxy‐3‐methylglutaryl‐CoA reductase
HMGCS1: 3‐hydroxy‐3‐methylglutaryl‐CoA synthase 1
H/R: hypoxia/reoxygenation
PE: preeclampsia
PFO: Perfringolysin O
PBS: phosphate buffered saline
LDL: low density lipoprotein
LDLR: low density lipoprotein receptor
MAPK: mitogen‐activated protein kinases
MTT: 3‐(4,5‐dimethylthiazol‐2‐yl)‐2,5‐diphenyltetrazolium bromide
n.d.: not determined
qRT‐PCR: quantitative reverse transcription‐PCR
ROS: reactive oxygen species
SR‐BI: scavenger receptor class B type 1
SREBPs: sterol regulatory element binding proteins
TC: total cholesterol
Tg: triglycerides
vWR: von Willebrand factor
YWHAZ: tyrosine 3‐monooxygenase/tryptophan 5‐monooxygenase

## 1 INTRODUCTION

Cardiovascular disease (CVD) is prevalent in the adult population and the leading cause of morbidity and mortality globally.^1^, ^2^ Pregnancy is a highly vulnerable period, where complications can influence maternal and fetal development, also potentially affecting future health in childhood and adulthood.^3^ Atherosclerosis, a progressive disease characterized by the formation of lipid plaques in the arteries and the dominant cause of CVD, may begin during intrauterine life, with early lesion formation in fetal vessels associated with hypercholesterolemia during pregnancy,^3^, ^4^, ^5^, ^6^, ^7^, ^8^, ^9^ adversely impacting later life. Preeclampsia (PE) is considered a risk factor for CVD later in the life of the mother and the offspring; it often involves inadequate blood flow to the placenta, leading to hypoxia or reduced oxygen supply to the fetus. Hypoxia in PE can have detrimental effects on both the mother and the developing fetus, potentially resulting in a range of complications.^10^ PE presents a proatherogenic maternal lipid profile and accumulation of cholesterol at the maternal–fetal interface.^11^, ^12^ In addition, in PE, there is a decrease in maternal plasma levels of high‐density lipoprotein (HDL), which has been associated with an increased risk of atherosclerosis.^13^, ^14^

Hypoxia has been identified as a common factor in several diseases, including atherosclerosis, ischemic heart disease,^15^, ^16^, ^17^ and as outlined above in pregnancy disorders such as PE and fetal growth restriction.^18^, ^19^, ^20^, ^21^ In the early stages of a healthy pregnancy, low oxygen (O_2_) availability (2%–3% or a PO_2_ of 15–20 mm Hg) is essential for progenitor cells, preventing DNA damage, as well as for the proliferation and differentiation process.^21^, ^22^ This hypoxic microenvironment rises at the end of the first trimester to “normal” physiological levels (8%–10% or a PO_2_ of 60–76 mm Hg), and this hypoxia/reoxygenation (H/R) process is regarded as physiological and essential for normal fetal and placental development.^23^ However, the harmful effects of hypoxia during pregnancy mostly occur during mid‐to‐late gestation, when prolongation of hypoxia or H/R events exists.^24^ It has been reported that long exposure to prenatal hypoxia results in impaired fetal endothelial function and fetal oxidative stress,^25^, ^26^ contributing to the development of hypertension, coronary heart disease, metabolic syndrome, and increased susceptibility to ischemic injury in humans.^27^, ^28^

Cholesterol plays a crucial role in fetal development as it is essential for the formation and proper functioning of the placenta.^29^, ^30^ The placenta, a vital structure during pregnancy, acts as a selective barrier that regulates the transport of nutrients, including cholesterol, from the mother to the fetus. However, hypoxia can have detrimental effects on this delicate transfer. Hypoxia can alter the expression of lipid transporters in the placenta and hinder the absorption of cholesterol by the fetus, which, in turn, may have negative consequences on fetal development.^31^, ^32^ Pregnancies suffering from PE have been described to exhibit a prolongation of hypoxic periods. The expression of the ATP‐binding cassette (ABC) transporter A1 (ABCA1), which is important in the regulation of lipid homeostasis by mediating cholesterol efflux,^33^, ^34^ was found to be reduced in placentae from women with PE.^35^ This could cause dysregulation in lipid transport, associated with an accumulation of cholesterol in the placental trophoblast layer in PE in both placentae^33^ and primary extravillous trophoblasts.^36^ Cholesterol efflux assays have shown that hypoxia significantly decreased efflux mediated by ABCA1 in primary macrophages,^37^ which may contribute to the progression of atherosclerotic lesions. These findings suggest that hypoxia can impact the risk of developing CVD in adults and alter cholesterol homeostasis in intrauterine life. However, the regulation of the mechanisms controlling cholesterol homeostasis during hypoxia and how this could affect the pathology of PE is not well understood.

In this study, we comprehensively investigated the effect of hypoxia on several crucial factors regulating cholesterol homeostasis in the human placenta using a well‐established model of primary human trophoblasts exposed to hypoxia and H/R, which mimics typical features of PE.^38^ We hypothesized that hypoxia alters cholesterol homeostasis in the placenta and thereby affects placental function during pregnancy. In this context, we determined intracellular cholesterol storage and esterification, cellular cholesterol biosynthesis and uptake, cholesterol conversion to progesterone, factors regulating gene expression, as well as cholesterol transport and efflux to maternal and fetal serum. Finally, to gain insight into the effects of hypoxia on the cellular regulation of cholesterol homeostasis, selected cellular signaling pathways were studied. Importantly, to assess whether this cell model reflects changes occurring in PE patients, we determined in parallel parameters reflecting intracellular cholesterol storage, cellular cholesterol biosynthesis, and gene expression of cholesterol efflux transporters in placentae from women who suffered from PE.

## 2 MATERIALS AND METHODS

### 2.1 Study group

Human placentae were collected in two countries (Switzerland and Chile). Healthy term pregnancies (38–40 weeks) were recruited at the Division of Gynecology and Obstetrics of the Lindenhofgruppe, Bern, Switzerland (*n* = 13), and the Hospital Clínico UC Christus, Chile (*n* = 14). Placentae from women with PE (32–40 weeks) were obtained solely from the Hospital Clínico UC Christus, Chile (*n* = 11). PE is defined as de novo hypertension (systolic blood pressure >140 mm Hg and diastolic blood pressure >90 mm Hg) after 20 weeks of gestation, accompanied by proteinuria and/or evidence of maternal acute kidney injury, liver dysfunction, neurological features, hemolysis or thrombocytopenia, or fetal growth restriction.^39^ PE was subclassified according to the manifestations and resolution of delivery: early‐onset PE (diagnosed before the 34th week of gestation) and late‐onset PE (diagnosed at or after the 34th week of gestation).^40^ For this study, only late‐onset PE samples were used.

All pregnant women were non‐smoking, did not consume alcohol or drugs, and were without intrauterine infection or other medical obstetrical complications. Women with multiple pregnancies, fetal growth restriction, and pre‐gestational and/or gestational diabetes mellitus were excluded from this study. The collection of samples was performed according to the principles outlined in the Declaration of Helsinki. All procedures were approved by the local ethics committee (Canton of Bern, Switzerland Basec Nr. 2016–00250) and the Faculty of Medicine at Pontificia Universidad Católica de Chile (PUC, ID‐180810004). Informed consent and clinical data from patients were obtained. General maternal (i.e., age, height, weight, and blood pressure) and neonatal (i.e., sex, gestational age, weight and height, and the weight of the placenta) variables were obtained from clinical records. The weight of the placenta was obtained only from those collected in Switzerland. The volume of the blood samples taken from women and umbilical cords was 20 mL and 10 mL, respectively. Due to restrictions regarding ethical approval, it was not possible to obtain blood samples from the cohort in Chile.

### 2.2 Determination of cholesterol in maternal and umbilical cord blood

Total cholesterol (TC), high density lipoprotein (HDL), low density lipoprotein (LDL), and triglyceride (Tg) levels were determined in maternal blood obtained from brachial venous blood and in umbilical cord blood at the time of delivery as described.^8^ TC, HDL, and Tg determination were measured by using standard enzymatic colorimetric assays (Cobas Integra) on a Cobas® 8000 modular analyzer series (Roche Diagnostic Corporation) at the Center for Laboratory Medicine Inselspital, Bern, Switzerland. LDL was calculated from TC, HDL, and Tg levels by applying Friedewald's equation.

### 2.3 Primary human trophoblast cells

Primary trophoblast cells were isolated from healthy term placental villous tissues. Placental tissue was carefully minced, and approximately 30 g was digested three times for 30 min at 37°C in saline Hanks/HEPES solution including DNase I (Sigma‐Aldrich, USA) and trypsin (Thermo Fisher Scientific, USA) as previously described.^41^ After digestion and centrifugation at 1000 RCF, the cellular pellets were resuspended in DMEM and separated by centrifugation (1500 RCF, 20°C, 20 min) using a 10%–70% Percoll gradient (Sigma‐Aldrich, USA). Trophoblast cells were obtained from Percoll gradient fractions between 35% and 55%. Cells were plated at a density of 200 000 cells/cm^2^ in DMEM high‐glucose (4.5 g/L glucose) medium supplemented with 10% fetal bovine serum and 1x antibiotic–antimycotic (Gibco, USA). After isolation, the cells were cultured for 12 h to allow them to attach before being exposed to different oxygen conditions.

The purity of the isolated trophoblast was evaluated by staining with the specific cell markers anti‐cytokeratin 7 (CK7), anti‐vimentin, or anti‐von Willebrand factor (vWF) (Novus Biologicals). Cells were acquired by flow cytometry (BD FACS LSRII; BD Biosciences, USA) as described.^38^ The purity of the isolated trophoblasts was 91%–96%.

### 2.4 Models of hypoxia and hypoxia/reoxygenation (H/R)

Trophoblast isolated from healthy term placentae were cultured as described above for 12 h and subsequently kept either at ambient O_2_ concentration, defined as a control condition and termed “normoxia” (5% pCO_2_, 21% pO_2_, 74% N_2_, 24 h), at low O_2_ concentration, termed “hypoxia” (5% pCO_2_, 1.5% pO_2_, 93.5% N_2_, 24 h) or H/R (6 h intervals alternating between normoxia and hypoxia until 24 h) in a hypoxic CO_2_ incubator (NU‐5731, NuAire, USA). The methodological procedures have been recently described in detail.^38^, ^42^

### 2.5 Isolation of mRNA and quantitative RT‐PCR

Total RNA was extracted both from placental tissue (approximately 100 mg obtained from PE and normotensive controls) and trophoblast cells after hypoxia/reperfusion using TRI reagent (Invitrogen, UK). Total RNA (1 μg) was reverse transcribed to cDNA using the GoScript™ Reverse Transcriptase System (Promega, USA) according to the manufacturer's instructions. Quantitative reverse transcription‐PCR (qRT‐PCR) was performed as previously described.^43^ In brief, qRT‐PCR was carried out on a CFX qRT‐PCR System using a SYBR® Green PCR master mix detection kit (Promega, USA). Primer pairs are listed in Table 1. The relative gene expression was calculated using the 2^−ΔΔCq^ method, using tyrosine 3‐monooxygenase/tryptophan 5‐monooxygenase (*YWHAZ*) as the reference gene.

**TABLE 1:** Nucleotide sequences of primers used for qRT‐PCR.

Gene	Primer sequence (5′‐3′)
*ABCG1*	F‐AACATGGAGGCCACTGAGAC R‐GGCCACCAACTCACCACTAT
*ABCA1*	F‐CCACATTTTTGCCTGGGACG R‐AGCGATTCTCCCCAAACCTT
*SR‐BI*	F‐CGGCTCGGAGAGCGACTAC R‐GGGCTTATTCTCCATGATCACC
*LDLR*	F‐GACGTGGCGTGAACATCTG R‐CTGGCAGGCAATGCTTTGG
*HMGCR*	F‐GATGGGAGGCCACAAAGAG R‐TTCGGTGGCCTCTAGTGAGA
*HMGCS1*	F‐AAGTCACACAAGATGCTACACCG R‐TCAGCGAAGACATCTGGTGCCA
*ACAT1*	F‐ATTCCTCTGCCTCTGCTGTC R‐AGACCAGAGAAACCCTGCAA
*SREBP‐1a*	F‐CGAAGACATGCTTCAGCTTATCA R‐CCAGCATAGGGTGGGTCAAA
*SREBP‐1c*	F‐TCGCGGAGCCATGGATT R‐GGAAGTCACTGTCTTGGTTGTTGA
*SREBP‐2*	F‐ CGGTAATGATCACGCCAACAT R‐TGGTATATCAAAGGCTGCTGGAT
*HIF‐2α*	F‐ ATGAAGAGCAAGCCTTCCAG R‐TGGGGTTTTGGGTGAACTTA
*HIF‐1α*	F‐CGTTGTGAGTGGTATTATTC R‐GGCTACTTGTATCTTCTGA
*YWHAZ*	F‐CCGTTACTTGGCTGAGGTTG R‐AGTTAAGGGCCAGACCCAGT

### 2.6 Western blotting

Placental tissue was homogenized to obtain protein extracts. Samples were lysed in solution 1 (10 mmol/L EDTA, 50 mmol/L Tris–HCl, pH 8.3), mixed with an equal volume of solution 2 (4% SDS, 20% glycerol, 125 mmol/L Tris/HCl, pH 6.8), heated (50°C, 10 min), sonicated (6 cycles, 10 s, 100 Watt, 4°C), and spun down (15 000 RCF, 20 min) as described.^44^, ^45^ Trophoblasts were lysed in protein extraction buffer (100 mmol/L NaCl, 0.5% Triton X‐100, 1% SDS, 50 mmol/L Tris–HCl, pH 7.4) containing a mixture of protease inhibitors (Sigma‐Aldrich, USA).

The protein concentration of the extracts was determined with the Pierce™ BCA Protein Assay Kit (Thermo Fisher Scientific, USA). Protein (30 μg for placental tissue and 40–60 μg for trophoblast cells) was separated by polyacrylamide gel electrophoresis (8%–10%) and transferred onto nitrocellulose membranes (BioRad Laboratories, Hertfordshire, UK). The protein for placental *tissue* was probed with primary *rabbit* polyclonal anti‐scavenger receptor class B type 1 (SR‐BI) (1 μg/mL), anti‐ABCG1 (1 μg/mL) (Novus Biological, USA), anti‐3‐hydroxy‐3‐methylglutaryl‐CoA synthase 1 (HMGCS) (1 μg/mL), and anti‐3‐hydroxy‐3‐methylglutaryl‐CoA reductase (HMGCR) (1 μg/mL) (Thermo Fisher Scientific, USA), as well as *mouse* monoclonal anti‐ABCA1 (1 μg/mL) and anti‐glyceraldehyde 3‐phosphate dehydrogenase (GAPDH) (0.2 μg/mL) (Santa Cruz Biotechnology Inc., USA) (18 h, 4°C). The protein for *trophoblast*s was probed with primary *rabbit* polyclonal anti‐SR‐BI (1 μg/mL), anti‐ABCA1 (1 μg/mL) (Novus Biological, USA), ABCG1 (2 μg/mL) (Genetex, USA), and anti‐P‐p44/42 mitogen‐activated protein kinases (MAPK) (1 μg/mL) (Cell Signaling Technology, USA) (18 h, 4°C) as well as primary *mouse* monoclonal anti‐total p44/42 MAPK (1 μg/mL) (Cell Signaling Technology, USA) (18 h, 4°C) and anti‐β‐actin (0.4 μg/mL, 1 h, room temperature) (Sigma‐Aldrich, USA) antibodies. After washing, the membranes were incubated with secondary horseradish peroxidase‐conjugated goat anti‐rabbit or anti‐mouse antibody (Thermo Fisher Scientific, USA) for placental tissues (1 h, room temperature, 0.2 μg/mL) or with IRDye® 680RD goat anti‐rabbit IgG secondary antibody and IRDye® 800CW goat anti‐mouse IgG secondary antibody (LI‐COR, USA) (2 h, room temperature, 0.05 μg/mL), respectively. Proteins were detected by enhanced chemiluminescence and quantified by densitometry. Uncropped blots are shown in the supplementary information (Figures S1–S9).

### 2.7 Immunofluorescence

Formalin‐fixed placental biopsies (10% buffered formalin solution, 24 h, 4°C) were processed for routine paraffin embedding and sectioning (5 μm) for histological analysis as previously described.^41^, ^45^ Sections were washed with phosphate buffered saline (PBS), then with PBS containing 0.1 mmol/L glycine (15 min, 20°C) and permeabilized with Triton X‐100 in PBS (0.5%, 1 h, room temperature). Briefly, the sections were incubated (1 h, room temperature) in a blocking buffer (300 mmol/L NaCl, 0.3% Triton X‐100, 0.2% fish gelatin, in PBS). Placental sections were incubated (18 h, 4°C, 10 μg/mL) with the primary antibodies for SR‐BI, ABCG1 (Novus Biological, USA), and ABCA1 (Santa Cruz Biotechnology Inc., USA). To detect unesterified cholesterol in the membrane, we used a recombinant perfringolysin O (PFO) fusion with Glutathione‐S‐Transferase (GST‐PFO) (1.5 h, room temperature, 5.8 μg/mL). After rinsing twice with blocking buffer, the secondary antibodies, that is, Alexa Fluor 488‐conjugated goat anti‐rabbit (H + L, λexc/λem: 495/568 nm, 3.33 μg/mL), Alexa Fluor 568‐conjugated goat anti‐mouse IgG (H + L, λexc/λem: 578/603 nm, 3.33 μg/mL) (Thermo Fisher Scientific, USA), or Alexa Fluor 594‐conjugated goat anti‐goat (H + L, λexc/ λem: 495/568 nm, 0.5 μg/mL) in blocking buffer containing 0.1 μg/mL DAPI (4′,6‐Diamidino‐2‐Phenylindole, Dihydrochloride) (Invitrogen, USA) were added.^45^ Tissue sections were cover slipped and incubated for 1 h at room temperature. After rinsing twice with PBS (room temperature), the coverslips were examined in a TCS SP8 laser‐scanning confocal microscope (Leica Microsystems, Wetzlar, Germany). Images were processed with ImageJ version 2.1.0 (NIH, USA). For the GST‐PFO, the fluorescence signal in trophoblasts was selected and corrected for the area and the background of the image. Negative controls are shown in the supplementary information (Figure S10).

### 2.8 Cholesterol efflux

The efflux of [^3^H]‐cholesterol from primary trophoblast cells to maternal or fetal serum was determined with minor modifications as previously described.^41^, ^46^ In brief, trophoblasts were cultured for 12 h and subsequently pre‐incubated for 24 h with DMEM containing 10% FBS supplemented with [^3^H]‐cholesterol (0.5 μCi/mL) under normoxia, hypoxia, and H/R conditions. After incubation, the culture medium was removed, and the cells were washed with PBS supplemented with bovine serum albumin‐free fatty acids (BSA‐FFA) (2 mg/mL). Subsequently, the cells were incubated with cholesterol acceptors (maternal and fetal serum; 5%, 6 h, 37°C). Thereafter, the culture medium was recovered, and the cells were lysed with 1 N KOH. Radioactivity was determined both in the culture medium and cell lysates, and the efflux was estimated as the fraction of radioactive signal in the medium compared to the total signal in the medium and cells as previously described.^41^

### 2.9 Measurement of intracellular cholesterol content

Folch extraction was performed on 50 μg of protein from trophoblast lysates as previously described.^41^, ^47^ Lysates were incubated with methanol/chloroform (1:2 v/v, 30 min, 50°C). Then, 1 volume of water was added (18 h, 4°C), and the reaction was centrifuged (750 rcf, 20 min, 4°C). The methanol/water phase was removed, and the chloroform phase containing cellular cholesterol was recovered and completely evaporated. Cholesterol extracted from trophoblasts was determined with the Cholesterol quantitation kit (Sigma‐Aldrich, USA) in the presence or absence of the enzyme cholesterol esterase for determination of total (TC) and non‐esterified cholesterol, respectively. Cholesteryl esters were calculated as the difference between TC and non‐esterified cholesterol.

### 2.10 Measurement of apolipoproteins and progesterone secretion by ELISA

The secretion of apolipoprotein A type I (ApoA‐I), apolipoprotein E (ApoE), and progesterone into the trophoblast culture media after 6 h to 24 h exposure to normoxia, hypoxia, and H/R was measured by using commercial ELISA kits (Human Apolipoprotein A1 ELISA^BASIC^ kit, No 3710‐1A, MABTECH, Sweden; ELISA Pro: Human ApoE, No 3712‐1HP‐1, MABTECH, Sweden; Human Progesterone Enzyme Immunoassay kit, RayBiotech, USA), following the manufacturer's instructions. Consecutive absorbance measurements were carried out at 450 nm on a Vmax microplate reader (Molecular device, USA). The concentrations of ApoA‐I, ApoE, and progesterone released by trophoblasts were calculated using the respective standard curves.

### 2.11 Detection of reactive oxygen species and cell viability assay

To quantify the intracellular formation of reactive oxygen species (ROS), primary trophoblasts exposed to normoxia, hypoxia, and H/R were loaded with 20 μM 2′,7′‐dichlorodihydrofluorescein diacetate (DCFH2‐DA) (MedChemExpress, USA) for 30 min at 37°C in 5% CO_2_. Subsequently, cells were washed twice with PBS and the fluorescence was measured at Ex/Em = 496/525 nm using a Flex Station II fluorescence microplate reader.

Cell viability was assessed using 3‐(4,5‐dimethylthiazol‐2‐yl)‐2,5‐diphenyltetrazolium bromide (MTT) (Sigma‐Aldrich, USA). Following each condition (normoxia, hypoxia, and H/R), cells were incubated with 20 μL of 5 mg/mL MTT stock solution in 100 μL of medium per well for 4 h at 37°C in 5% CO_2_. Subsequently, the medium was removed, and 150 μL of dimethyl sulfoxide was added. After thorough mixing in darkness at room temperature for 10 min, the absorbance was measured on a microplate reader at 570 nm.

### 2.12 Statistical analyses

Values for maternal and neonatal characteristics are presented as mean ± SD. The relative gene and protein expression, protein/progesterone secretion, cholesterol content, and cholesterol efflux percentages are presented as the mean ± SEM for the experiments, where n indicates either the number of different placentae from which primary trophoblasts were isolated and cultured or the number of individual placentae investigated. Two or more groups were compared using the Mann–Whitney test and ANOVA, respectively. *p ≤ .05, **p ≤ .01, ***p ≤ .001, and ****p ≤ .0001 were considered statistically significant. GraphPad Prism 9.5.0 (GraphPad Software Inc., USA) was used to analyze the data and prepare the graphs.

## 3 RESULTS

### 3.1 Maternal and neonatal variables

The clinical characteristics of the women providing placentae for the cell culture experiments and the biochemical analysis of healthy versus PE pregnancies are shown in Table 2. All the clinical variables for the mothers and the newborns from which the placentae were used to isolate trophoblasts and to perform cell culture experiments were in the normal range. Maternal cholesterol levels were considered physiological for pregnancy according to previously published standards.^48^ Women with PE showed an increase in systolic and diastolic blood pressure compared to healthy pregnancies. Creatinine and proteinuria in PE were highly elevated. The gestational age in PE was reduced compared to healthy pregnancies (Table 2). Due to restrictions regarding ethical approval, it was not possible to obtain blood samples from the patient cohort in Chile. Therefore, data regarding the lipid profile in maternal and cord blood from the patients in Chile are lacking and are indicated as not determined (n.d). Likewise, data concerning the placental weight in the healthy and PE cohorts from Chile were not available.

**TABLE 2:** Clinical characteristics of pregnant women and newborns.

	Samples for trophoblast isolation/ culturing (Switzerland)	Samples for comparison preeclamptic versus healthy placentae (Chile)	
Type of pregnancy	Control	Healthy	PE
Sample size	*n* = 13	*n* = 14	*n* = 11
*Maternal variables*			
Age (years)	32.70 ± 5.60	30 ± 4.4	30.8 ± 3.6
Length (cm)	167 ± 6.85	161.2 ± 6.2	159.1 ± 7.5
Weight pre‐pregnancy (kg)	68.36 ± 12.04	58.4 ± 6.1	59.4 ± 11.5
Weight at delivery (kg)	79.37 ± 12.26	69.6 ± 6.3	72.9 ± 11.8
BMI pre‐pregnancy (kg/m^2^)	23.85 ± 3.00	22.5 ± 1.8	23.41 ± 4.2
BMI at delivery (kg/m^2^)	28.18 ± 2.89	26.8 ± 1.5	28.43 ± 4.1
Systolic blood pressure at delivery (mm Hg)	117.30 ± 11.24	111.5 ± 8.0	156.5 ± 17.8*
Diastolic blood pressure at delivery (mm Hg)	74.42 ± 8.94	70.2 ± 4.9	91.6 ± 9.3*
Creatinine (mg/dL)	n.d.	n.d.	108.4 ± 81.3
Proteinuria (mg/dL)	n.d.	n.d.	2839 ± 5669
Total cholesterol (mg/dL)	261.7 ± 41.53	n.d.	n.d.
Triglycerides (mg/dL)	239.5 ± 69.19	n.d.	n.d.
HDL (mg/dL)	64.66 ± 12.23	n.d.	n.d.
LDL (mg/dL)	143.3 ± 37.77	n.d.	n.d.
*Newborn variables*			
Sex (female/male)	7/6	8/6	7/4
Placenta weight	694.1 ± 116.5	n.d.	n.d.
Gestational age (weeks)	38.5 ± 0.57	39.2 ± 0.8	36.9 ± 2.4*
Route of delivery (C‐section/labor)	13/0	7/7	8/3
Birthweight (g)	3499 ± 346.7	3381 ± 304.7	2865 ± 923.6 (*p* = .06)
Length (cm)	50.69 ± 1.65	50.4 ± 1.8	47.9 ± 5.1
Ponderal index (g/cm^3^ × 100)	2.69 ± 0.27	2.6 ± 0.2	2.5 ± 0.2
Total cholesterol (mg/dL)	66.61 ± 12.27	n.d.	n.d.
Triglycerides (mg/dL)	36.75 ± 9.39	n.d.	n.d.
HDL (mg/dL)	36.03 ± 9.37	n.d.	n.d.
LDL (mg/dL)	21.28 ± 7.53	n.d.	n.d.

*Note*: The weight and body mass index (BMI) were determined pregestationally and in the third trimester; blood pressure and lipid profile were determined at delivery.

Abbreviations: HDL, high density lipoprotein; LDL, low density lipoprotein; PE, preeclampsia; n.d., not determined.

* *p* < .05 pregnancies without preeclampsia (healthy) versus the corresponding values in the preeclamptic group. Data are presented as mean ± SD.

### 3.2 Exposure to hypoxia alters genes involved in cholesterol synthesis in primary trophoblasts

**FIGURE 1:**
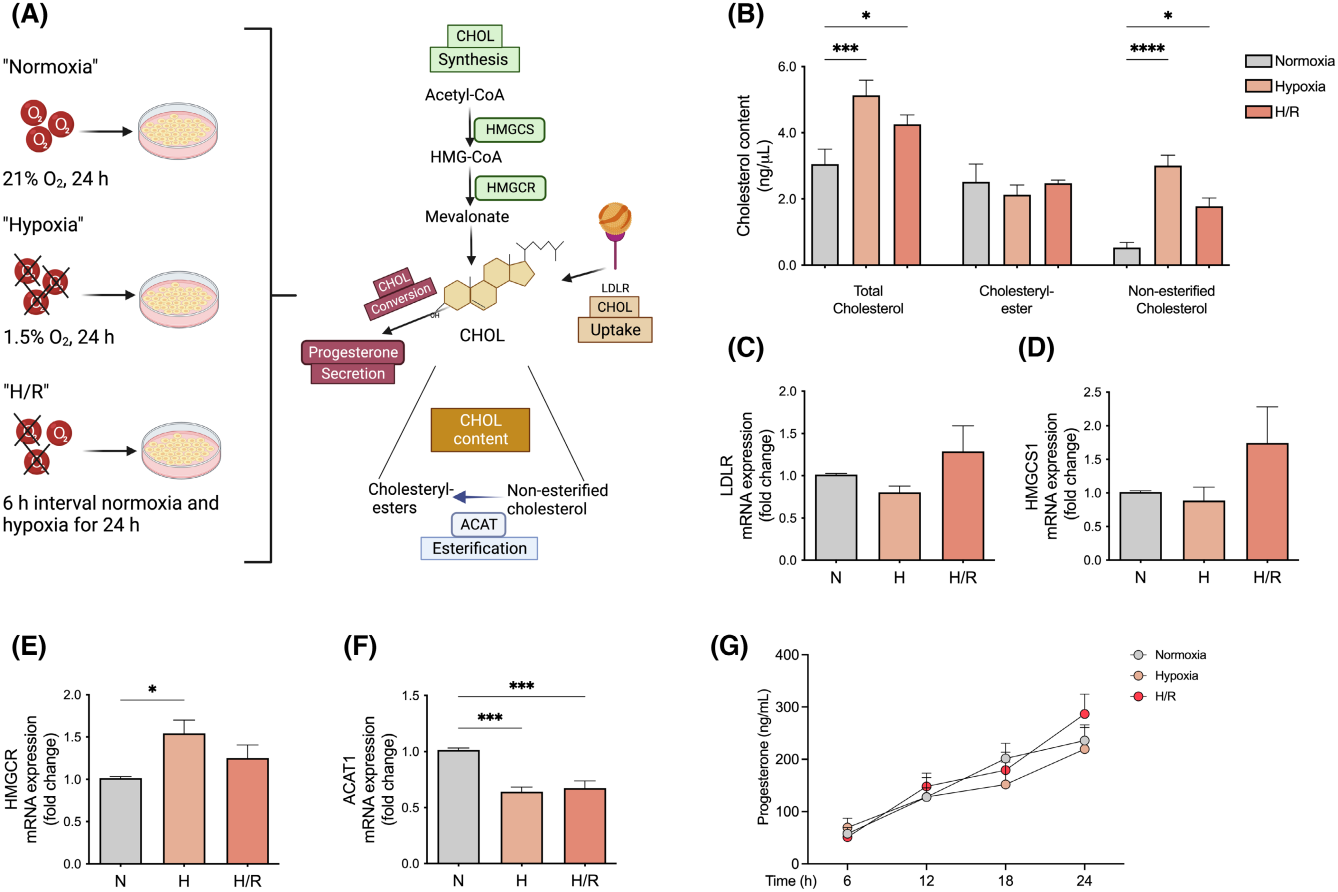
Cholesterol homeostasis pathways are disturbed in primary trophoblasts after hypoxic insult. (A) This figure provides an overview of different pathways and factors involved in the regulation of cholesterol levels in the human placenta. Cholesterol biosynthesis involves the key enzymes HMGCS1 and HMGCR, while the LDL receptor is important for cholesterol uptake and the maintenance of intracellular cholesterol levels. Cholesterol is not only synthesized or taken up in the trophoblast but can also be included in cell membranes or intracellularly stored as cholesteryl esters. Alternatively, it can be converted to other steroid hormones such as progesterone, which is vital for maintaining pregnancy. In summary, the figure demonstrates the impact of hypoxia on cholesterol regulation in the trophoblast, including synthesis, uptake, storage, and conversion into other essential bioactive molecules. (B) Cellular cholesterol content was determined using an enzymatic reaction‐based assay. The content of total cholesterol, cholesteryl esters, and non‐esterified cholesterol was measured in 50 μg protein of cell lysates. (C–F) The mRNA expression of (C) LDLR, (D) HMGCS1, (E) HMGCR, and (F) ACAT1 was determined by real‐time quantitative RT‐PCR in trophoblast cells and normalized to the reference gene YWHAZ. Values represent the fold change (2^−ΔΔCq^) of the mRNA expression in hypoxia (H) or hypoxia/reoxygenation (H/R) as compared to normoxia (N). (G) Secretion of progesterone was determined by ELISA in supernatants of primary trophoblast cells. Trophoblasts were exposed either to normoxia (N; 24 h; gray bar), hypoxia (H; 24 h; light orange), or hypoxia/reoxygenation (H/R; 6 h intervals each of normoxia and hypoxia for 24 h; dark orange). Data are shown as the mean ± SEM, *n* = 7 per group. For comparison of normoxia with hypoxia and H/R, ANOVA was applied. *p < .05, **p < .01, ***p < .001 versus normoxia and versus 6 h in progesterone. Figure 1A was created with BioRender.com. ACAT1, acetyl‐CoA acyltransferase 1; CHOL, cholesterol; HMGCR, 3‐hydroxy‐3‐methylglutaryl‐CoA reductase; HMGCS1, 3‐hydroxy‐3‐methylglutaryl‐CoA synthase 1; LDLR, low‐density lipoprotein (LDL) receptor; YWHAZ, tyrosine 3‐monooxygenase/tryptophan 5‐monooxygenase.

To determine whether hypoxia overall influences cholesterol homeostasis, we analyzed the effect of hypoxia on cholesterol uptake, intracellular cholesterol synthesis, and storage, as well as the conversion of cholesterol to the crucial pregnancy hormone progesterone (Figure 1A).

We first evaluated the intracellular total and non‐esterified cholesterol content using an enzymatic reaction‐based assay. Intracellular total cholesterol levels were significantly increased in hypoxia and H/R (1.68‐ and 1.4‐fold respectively; *p* ≤ .001 and *p* ≤ .05, respectively; Figure 1B). Similarly, and even more pronounced, non‐esterified cholesterol was elevated after 24 h in hypoxia and H/R (6.16‐ and 4.39‐fold, respectively; *p* ≤ .0001 and *p* ≤ .05, respectively; Figure 1B), while the content of cholesteryl esters remained unchanged (*p* > .05; Figure 1B).

To determine whether the increased total cholesterol levels were due to an enhanced receptor‐mediated cholesterol uptake, we analyzed the mRNA expression of the LDL receptor (LDLR); no differences between the groups were found (*p* > .05; Figure 1C). To investigate whether the intracellular accumulation of cholesterol under conditions of hypoxia and H/R was related to altered cholesterol biosynthesis, we measured the mRNA expression of HMGCR and HMGCS1, the rate‐limiting enzyme for cholesterol biosynthesis and metabolic conversion of acetyl‐CoA, respectively. While no differences in mRNA expression were detected for HMGCS1 (*p* > .05; Figure 1D), HMGCR was significantly (*p* ≤ .05) increased (1.5 ± 0.16 vs. 1.01 ± 0.02, fold change; Figure 1E), in hypoxia compared with normoxia. On the other side, the intracellular accumulation of non‐esterified cholesterol was associated with a significant (*p* ≤ .001) reduction in the mRNA expression of ACAT1, an enzyme catalyzing the esterification of non‐esterified cholesterol, under hypoxic and H/R conditions (0.64 ± 0.04 and 0.67 ± 0.06 vs. 1.01 ± 0.02, fold change, respectively; Figure 1F).

Finally, to determine whether the increased synthesis of cholesterol, the precursor for other steroids, may affect the secretion of progesterone, we analyzed the progesterone concentration in the supernatants of the cells exposed to normoxia, hypoxia, and H/R. A continuous increase in progesterone secretion during 24 h was detected in all three groups, without significant differences between hypoxia and H/R compared with normoxia (Figure 1G).

### 3.3 Transcription factors and signaling pathways controlling cholesterol homeostasis are regulated by hypoxia in primary trophoblasts

**FIGURE 2:**
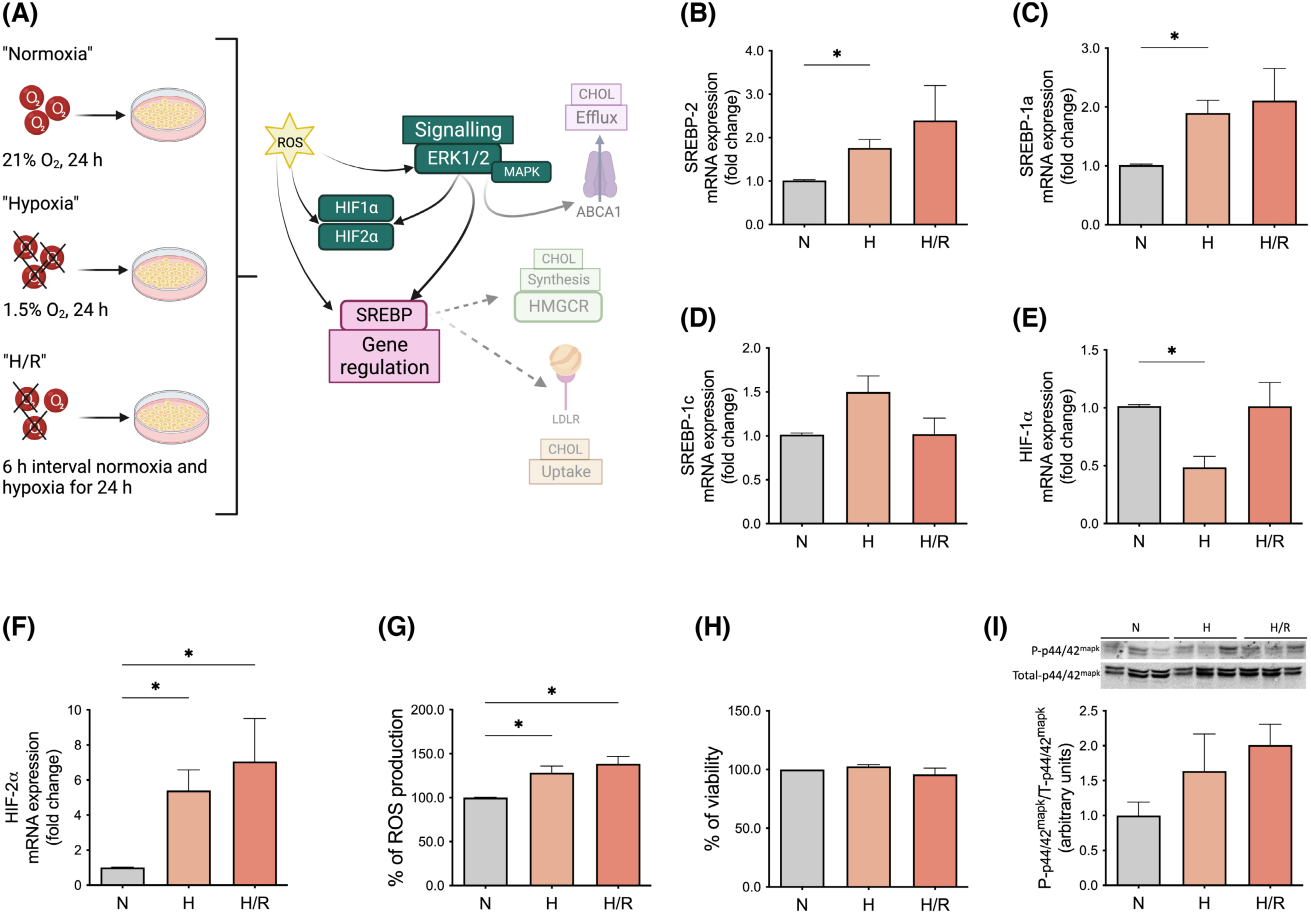
Effects of hypoxia on transcription factors, ROS production, cell viability, and signaling pathways regulating cellular cholesterol homeostasis. (A) For studying the impact of hypoxic conditions on the regulation of cholesterol levels, key components controlling cholesterol homeostasis were explored as shown in panels B to I. The focus was on understanding the roles of transcription factors like SREBP, HIF, relevant signaling pathways (MAPK), and the generation of reactive oxygen species (ROS). ROS serve as secondary messengers, influencing the activation and responses of SREBP, HIF, and MAPK pathways. These interactions are vital for comprehending the impact of reduced oxygen levels on lipid metabolism, cellular responses, and the broader implications of hypoxia on cholesterol homeostasis. (B–F) The mRNA expression of (B) SREBP‐2, (C) SREBP‐1a, (D) SREBP‐1c, (E) HIF‐1α and (F) HIF‐2α, (G) ROS production, (H) cell viability and protein levels of (I) MAPK total and phosphorylated (total‐p44/42^mapk^/P‐p44/42^mapk^). mRNA was determined by real‐time quantitative RT‐PCR in primary trophoblast cells and normalized to the reference gene YWHAZ. Values represent the fold change (2^−ΔΔCq^) of the mRNA expression in hypoxia (H) and hypoxia/reoxygenation (H/R) compared to normoxia (N). Protein expression was determined by Western blot analysis. Cell viability was evaluated using an MTT‐based assay as described in the section Methods. Trophoblasts were exposed either to normoxia (N; 24 h; gray bar), hypoxia (H; 24 h; light orange), andH/R (6 h intervals each of normoxia and hypoxia for 24 h; dark orange). Data are shown as mean ± SEM, *n* = 6–7 per group. For comparison of normoxia with hypoxia and H/R, ANOVA was applied. *p < .05, **p < .01, ***p < .001 versus normoxia. Figure 2A was created with BioRender.com. HIF, hypoxia‐inducible factor; MAPK, mitogen‐activated protein kinases (ERK1/2: the extracellular‐signal‐regulated kinase); MTT, 3‐(4,5‐dimethylthiazol‐2‐yl)‐2,5‐diphenyltetrazolium bromide; ROS, reactive oxygen species; SREBPs, sterol regulatory element binding proteins; YWHAZ, tyrosine 3‐monooxygenase/tryptophan 5‐monooxygenase.

We further evaluated in this trophoblast model central factors involved in the regulation of lipid availability and supply, such as the membrane‐bound sterol regulatory element‐binding protein (SREBP) transcription factors, the hypoxia‐inducible factors (HIF), ROS production, and underlying MAPK‐related signaling pathways controlling cholesterol homeostasis (Figure 2A).

SREBP‐2 and SREBP‐1a, known to control the supply of cholesterol and fatty acids,^49^ respectively, were increased (*p* ≤ .05) in both hypoxic conditions compared with normoxia (1.76 ± 0.2 and 1.90 ± 0.2 vs. 1.01 ± 0.02, fold change, respectively; Figure 2B,C). The mRNA expression of SREBP‐1c in trophoblasts exposed to hypoxia or H/R was not significantly different from controls (*p* > .05; Figure 2D). We also evaluated the mRNA expression of HIF‐1α and HIF‐2α in the trophoblasts at normoxia, hypoxia, and H/R since these were reported to be involved in the maintenance of the cellular lipid supply.^50^, ^51^ While the mRNA abundance of HIF‐1α was significantly reduced in hypoxia compared to normoxia (0.49 ± 0.1 vs. 1.01 ± 0.2, fold change; *p* ≤ .05; Figure 2E), HIF‐2α levels were higher in both hypoxia (approx. 5‐fold) and H/R (approx. 7‐fold), (*p* ≤ .05; Figure 2F).

To investigate whether hypoxic conditions resulted in increased oxidative stress, we measured the production of ROS in cells following exposure to hypoxia and H/R. Our data revealed a substantial increase in ROS levels in cells subjected to hypoxia and H/R when compared to cells maintained under normoxic conditions (128 ± 7.7 vs. 138 ± 8.6 vs. 100 ± 0.6%; *p* ≤ .05; Figure 2G). To rule out that the increased intracellular stress induced by hypoxia resulted in increased cell death, we determined cell viability by using an MTT‐based assay. No significant differences between normoxia and hypoxic conditions were found (Figure 2H).

To study underlying signaling pathways controlling cholesterol homeostasis, we evaluated the effects of hypoxia on the MAPK signaling pathway since the latter was shown to be involved in the phosphorylation of SREBP‐2 and in regulating cholesterol efflux.^52^ However, protein levels of total and phosphorylated p44/42 MAPK, as well as the ratio between the phosphorylated and total (P‐p44/42/total p44/42) MAPK, were not significantly different between normoxia, hypoxia, and H/R in the trophoblast cells (*p* > .05; Figure 2I). However, the Western blot data seem to suggest that the hypoxic environment provokes a tendency toward increased P‐p44/42/total p44/42 MAPK levels (Figure 2I).

### 3.4 Cholesterol efflux in primary trophoblasts is increased after exposure to hypoxia and H/R

**FIGURE 3:**
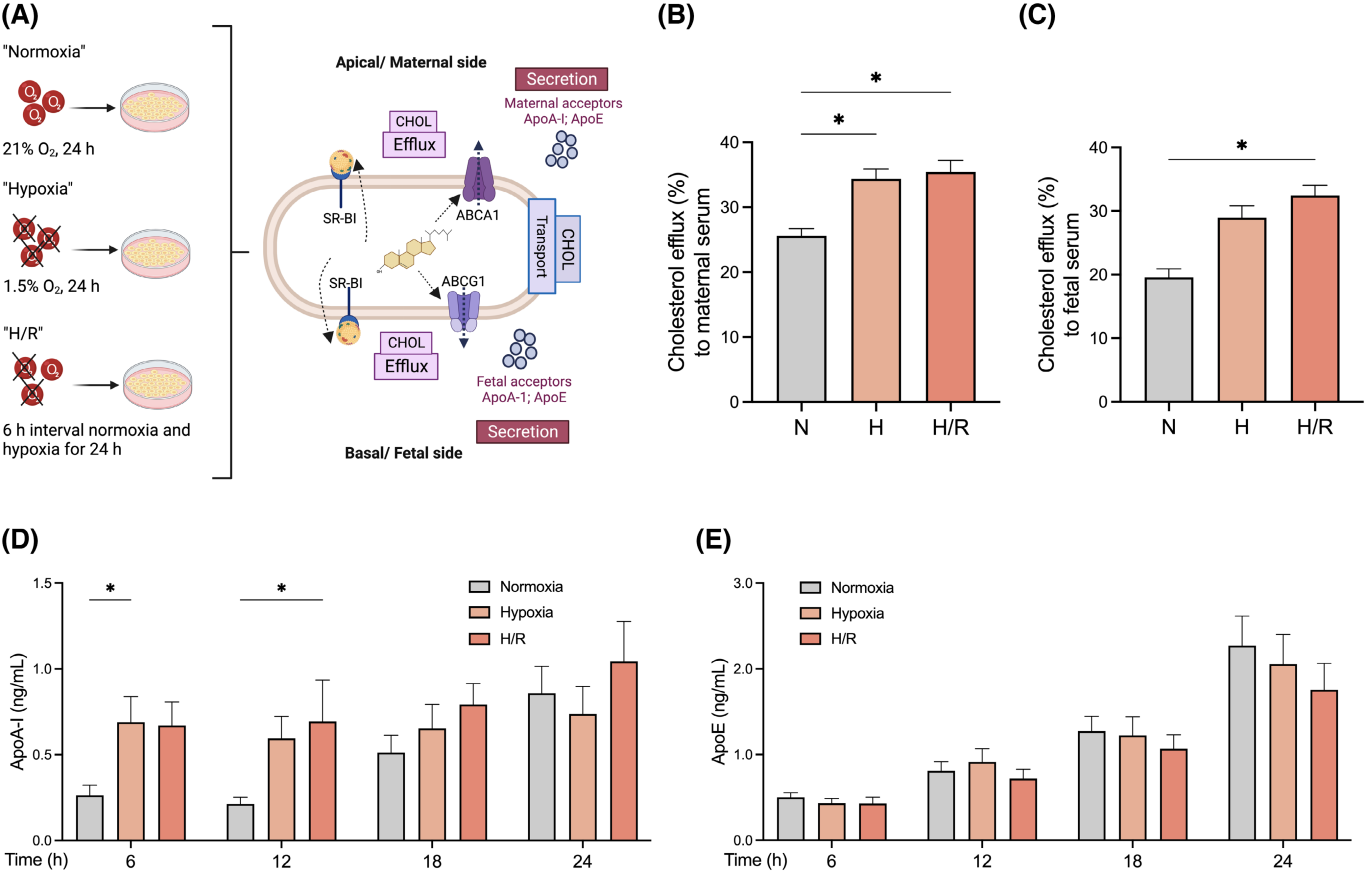
Cholesterol efflux to maternal and fetal serum is increased in primary trophoblasts exposed to hypoxia. (A) Figure 3 focuses primarily on two key aspects impacting intracellular cholesterol (CHOL) levels: cholesterol efflux and—related to this—apolipoprotein (Apo) secretion since apolipoproteins are extracellular acceptors for effluxed cholesterol. The results shown in panels B–E provide insights into the interplay between these pathways and demonstrate the effects of hypoxia on the balance of intra‐ and extracellular cholesterol levels and the release of apolipoproteins. Cholesterol efflux to (B) maternal and (C) fetal serum (5%, 6 h, 37°C) was assessed in primary trophoblast as described in the section Materials and Methods. Trophoblast cell secretion of (D) ApoA‐I and (E) ApoE was determined by ELISA in the supernatants of trophoblast between 6 h and 24 h. Trophoblasts were exposed either to normoxia (N; 24 h; gray bar), hypoxia (H; 24 h; light orange), or hypoxia/reoxygenation (H/R; 6 h intervals each of normoxia and hypoxia for 24 h; dark orange). Data are shown as mean ± SEM, *n* = 7 per group. For comparison of normoxia with hypoxia and H/R, ANOVA was applied. *p < .05, **p < .01, ***p < .001 versus normoxia. Figure 3A was created with BioRender.com.

To investigate whether the trophoblast cells respond to the increased intracellular synthesis and accumulation under hypoxia (see Figure 1), with alterations in cholesterol export, we performed cholesterol efflux experiments using maternal and fetal serum which naturally contain cholesterol acceptors. Cholesterol efflux is mainly mediated by the cholesterol transporters ABCA1, ABCG1, and SR‐BI (Figure 3A).

Cholesterol efflux to maternal serum was increased after exposure to hypoxia and H/R compared to normoxia (34.37 ± 1.52 and 35.44 ± 1.76 vs. 25.60 ± 1.12%, respectively; *p* ≤ .05; Figure 3B). Similarly, cholesterol efflux to fetal serum was also higher in H/R compared to normoxia (32.43 ± 1.61 vs. 19.55 ± 1.34%, respectively; *p* ≤ .05; Figure 3C). A similar, however not significant trend (P = 0.068) of higher cholesterol efflux rates toward fetal serum was found in the hypoxia condition.

To determine whether the increased cholesterol efflux during hypoxia may be related to an enhanced secretion of cholesterol acceptors by trophoblasts, we measured by ELISA the concentrations of ApoA‐I and ApoE in the cell supernatants between 6 h and 24 h. Hypoxia and H/R initially promoted the protein secretion of ApoA‐I at 6 h and 12 h compared to normoxia but had no persistent effect at later time points (Figure 3D). ApoE secretion remained unaffected at all measured time points (Figure 3E).

### 3.5 Cholesterol transporters in primary trophoblasts are altered by hypoxia

**FIGURE 4:**
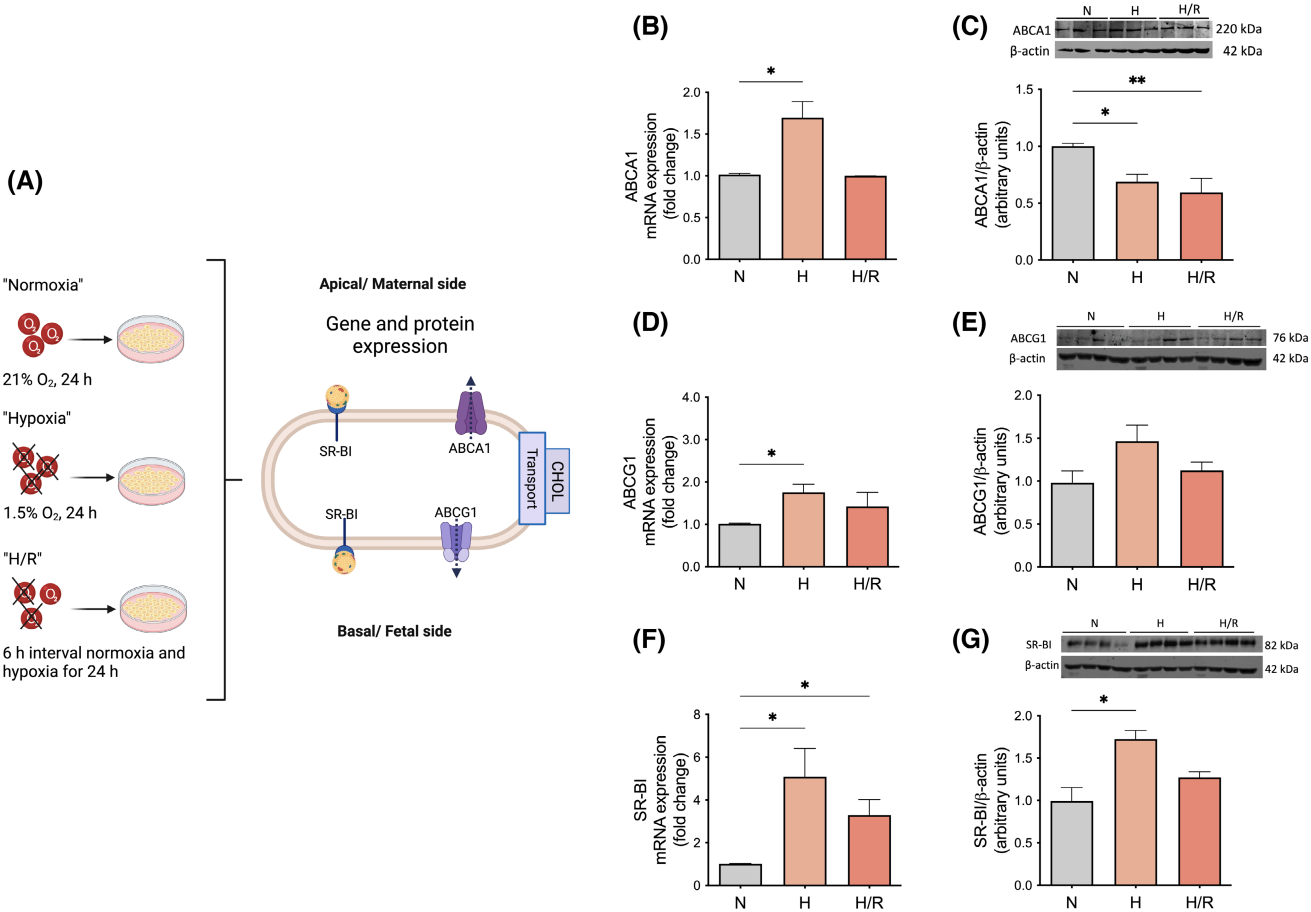
The cholesterol transporters ABCA1, ABCG1, and SR‐BI are altered in primary trophoblasts exposed to hypoxia. (A) This illustration gives an overview of the pathways and parameters delineated in panels B‐G, where the effect of hypoxic conditions on the intricate network of cholesterol transporters was investigated. (B–G) The mRNA and protein expressions of (B and C) ABCA1, (D and E) ABCG1, and (F and G) SR‐BI are shown. mRNA was determined by real‐time quantitative RT‐PCR in trophoblast cells and normalized to the reference gene YWHAZ. Values represent the fold change (2^−ΔΔCq^) of the mRNA expression in hypoxia (H) or hypoxia/reoxygenation (H/R) compared to normoxia (N). Protein expressions were determined by Western blot analysis using β‐Actin as a reference (loading control). Trophoblasts were exposed either to normoxia (N; 24 h; gray bar), hypoxia (H; 24 h; light orange), or H/R (6‐h intervals each of normoxia and hypoxia for 24 h; dark orange). Data are shown as mean ± SEM, *n* = 6–7 per group. For comparison of normoxia with hypoxia and H/R, ANOVA was applied. *p < .05, **p < .01, ***p < .001 versus normoxia. Figure 4A was created with BioRender.com. ABCA1, ATP‐binding cassette (ABC) transporter A1; ABCG1, ATP‐binding cassette (ABC) transporter G1; CHOL, cholesterol; SR‐BI, scavenger receptor class B type 1; YWHAZ, tyrosine 3‐monooxygenase/tryptophan 5‐monooxygenase.

To study whether the observed changes in cholesterol efflux are related to alterations in the expression of cholesterol transporters, we evaluated the effect of hypoxic conditions on the mRNA and protein expression of ABCA1, ABCG1, and SR‐BI in trophoblasts (Figure 4A). The mRNA abundance of ABCA1 was increased by hypoxia compared to normoxia (1.7 ± 0.29 vs. 1.01 ± 0.01, fold change, respectively; *p* ≤ .05; Figure 4B). In contrast to the mRNA expression results, the protein levels of ABCA1 were reduced in the hypoxia and H/R group compared to normoxia (0.69 ± 0.09 and 0.59 ± 0.09 vs. 1.01 ± 0.08, arbitrary units; *p* ≤ .05 and *p* ≤ .01, respectively; Figure 4C).

The mRNA expression of ABCG1 was increased by hypoxia compared to normoxia (1.76 ± 0.19 vs. 1.01 ± 0.01, fold change, respectively; *p* ≤ .05; Figure 4D). The protein abundance of ABCG1 was not significantly different (Figure 4E), but the trend paralleled the pattern found in the mRNA expression analysis (Figure 4D).

SR‐BI was affected by both hypoxia and H/R conditions compared to normoxia (5.08 ± 1.33 and 3.28 ± 0.73, fold change, vs. 1.01 ± 0.01, respectively; Figure 4F). SR‐BI protein was increased in hypoxia compared to normoxia (1.72 ± 0.1 vs. 0.99 ± 0.16, arbitrary units, respectively; *p* ≤ .05; Figure 4G), following the expression pattern found in the mRNA.

### 3.6 Cholesterol synthesis is altered in PE placental tissues

**FIGURE 5:**
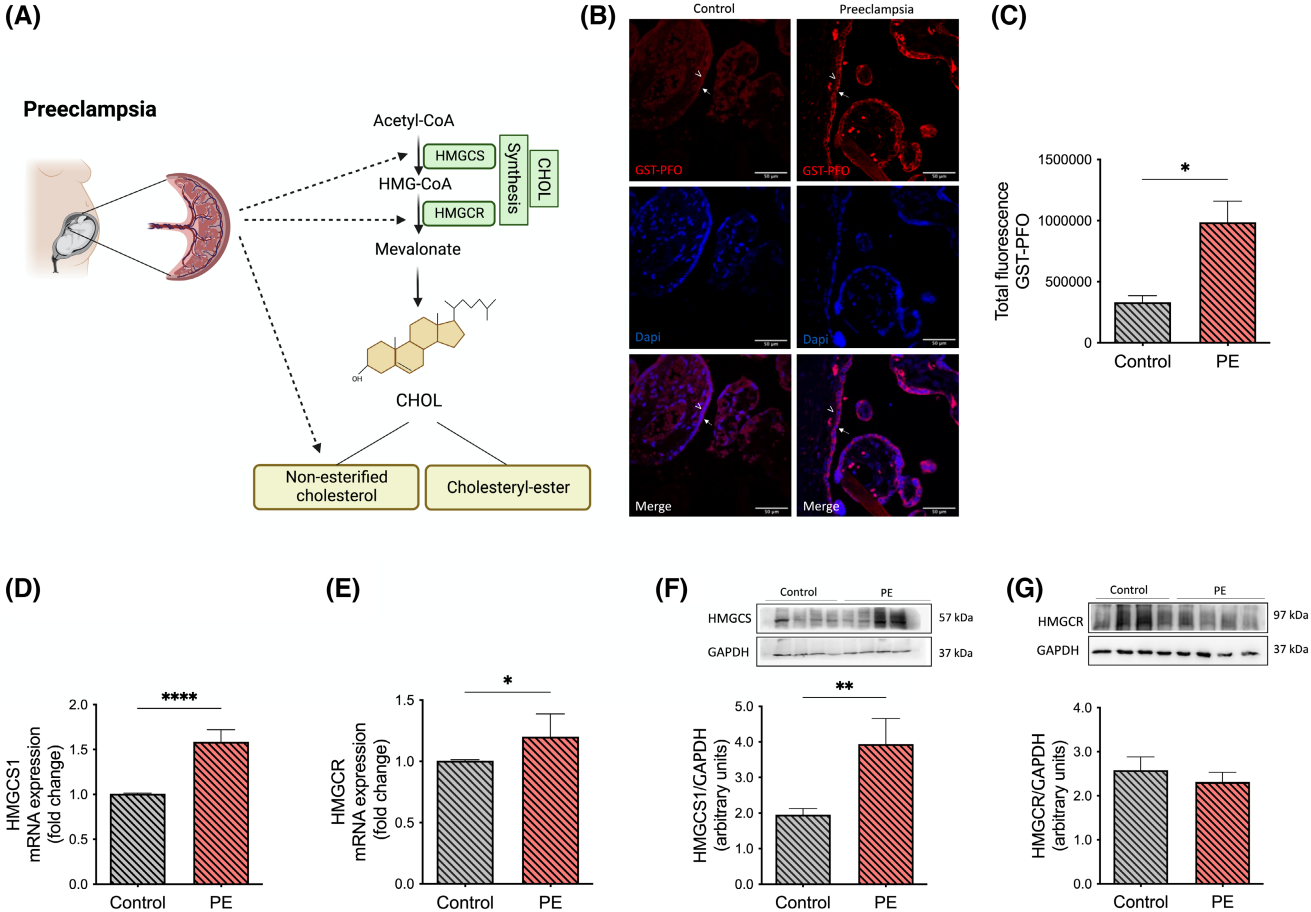
Parameters of cholesterol homeostasis are altered in preeclamptic placentae. (A) This figure provides an overview of the pathways and parameters depicted in panels B–G, which were systematically investigated to assess the impact of preeclampsia on cholesterol homeostasis. The emphasis of Figure 5 is put on mechanisms regulating cholesterol biosynthesis and storage. (B) Intracellular cholesterol visualized in human placentae obtained from PE patients and controls by staining with a recombinant PFO fusion with GST (GST‐PFO) (red) (arrow: apical membrane, arrowhead: basal membrane of trophoblasts). Nuclei are stained with Dapi (blue). Scale bar: 50 μM. (C) Semi‐quantitative analysis of the GST‐PFO signal found in trophoblast cells, where the fluorescence signal in trophoblasts within the tissue was selected and corrected for the area and the background. (D and E) The mRNA expression of (D) HMGCS1 and (E) HMGCR were determined by real‐time quantitative RT‐PCR in placental tissue and normalized to the reference gene YWHAZ. Values represent the fold change (2^−ΔΔCq^) of the mRNA expression found in PE placentae in comparison to controls. (F and G) Protein expression of (F) HMGCS1 and (G) HMGCR was analyzed by Western blotting using GAPDH as a reference. Data are shown as mean ± SEM, *n* = 6–14 (control; gray bar) and *n* = 6–11 (PE; orange bar). Immunofluorescence of GST‐PFO is shown for a representative image taken from a total of three analyzed placentae per group. Student's t‐test was applied to compare the control and PE placentae. *p < .05, **p < .01, ****p < .0001. Figure 5A was created with BioRender.com. CHOL, cholesterol; Dapi, 4′,6‐Diamidino‐2‐Phenylindole, Dihydrochloride; GAPDH, glyceraldehyde 3‐phosphate dehydrogenase; GST, glutathione‐S‐transferase; HMGCS1, 3‐hydroxy‐3‐methylglutaryl‐CoA synthase 1; HMGCR, 3‐hydroxy‐3‐methylglutaryl‐CoA reductase; PE, preeclampsia; PFO, perfringolysin O; YWHAZ, tyrosine 3‐monooxygenase/tryptophan 5‐monooxygenase.

Since (i) we found several parameters of cholesterol homeostasis affected by hypoxia in the trophoblast model of PE (see above) and (ii) hypoxia and/or H/R episodes are discussed as a mechanism contributing to PE, we further evaluated selected aspects of cholesterol homeostasis in preeclamptic versus control placentae (Figure 5A).

With a recombinant GST‐PFO probe, we determined non‐esterified (free) cholesterol in placental tissue sections and found that in preeclamptic placentae, non‐esterified cholesterol detected in trophoblast cells was more intense compared to controls (Figure 5B). By quantifying the signal obtained in the trophoblasts and correcting for the area and background, the results revealed an accumulation of non‐esterified cholesterol in placental tissue from women who had PE (Figure 5C).

In parallel to the trophoblast model, we also evaluated the mRNA and protein expression of HMGCS and HMGCR in placental tissues from PE patients and controls. The mRNA abundance of HMGCS and HMGCR was increased in the placentae of women who had PE compared to controls (1.59 ± 0.14 and 1.2 ± 0.19 vs. 1.01 ± 0.01, fold change; *p* ≤ .0001, *p* ≤ .05, respectively; Figure 5D,E). This increase was also confirmed on protein level for HMGCS (*p* ≤ .01; Figure 5F), but not for HMGCR (Figure 5G).

### 3.7 Expression of cholesterol transporters is altered in PE placental tissue

**FIGURE 6:**
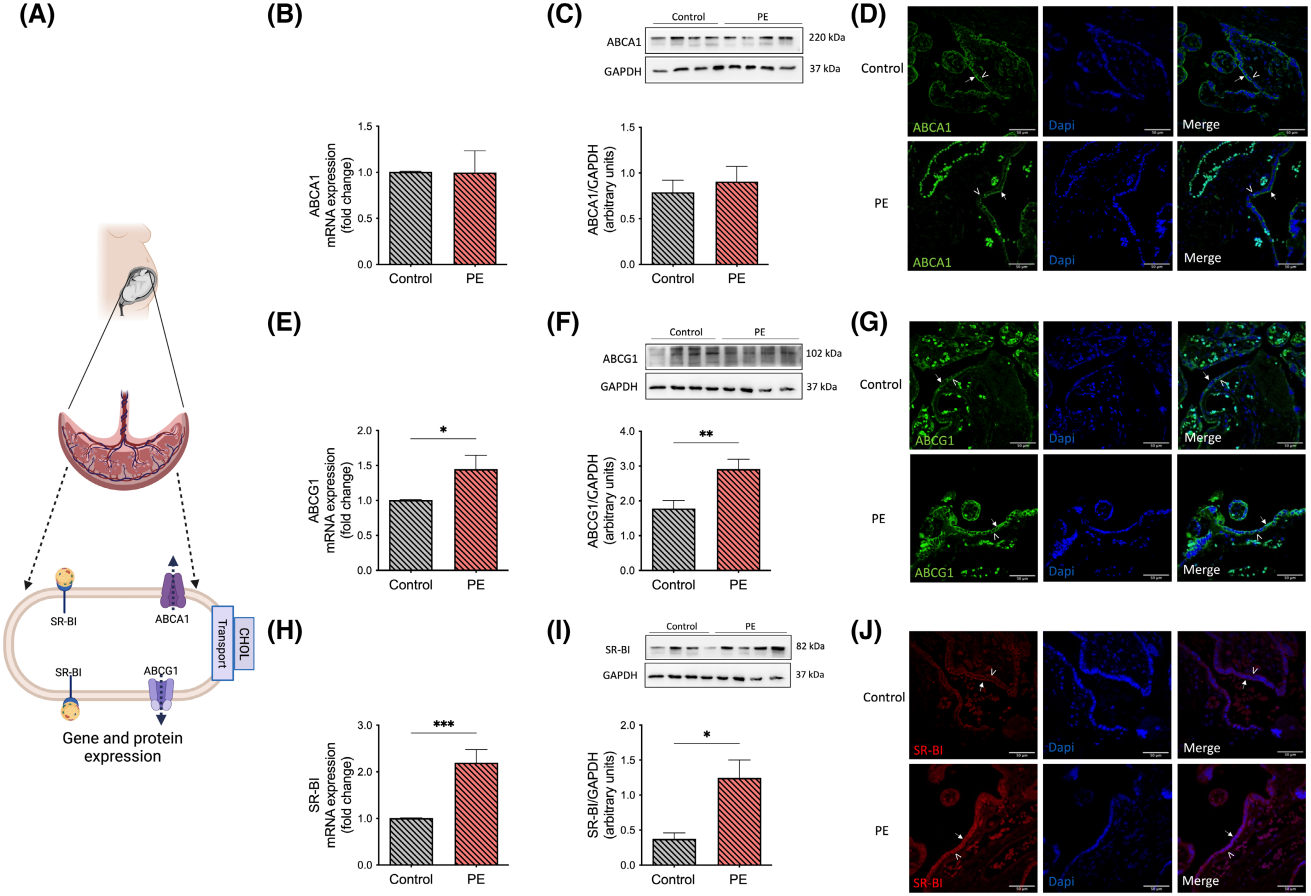
Cholesterol transporters ABCA1, ABCG1, and SR‐BI are altered in preeclamptic placental tissues. (A) This section provides a comprehensive overview of the pathways and parameters delineated in panels B–J, which were studied to assess the influence of preeclampsia (PE) on cholesterol homeostasis. Figure 6 is primarily focused on the tight regulation of cholesterol transporters, including ABCG1, ABCA1, and SR‐BI, and aims to elucidate their expression patterns in the context of PE. (B–J) The mRNA and protein expression of (B–D) ABCA1, (E–G) ABCG1, and (H–J) SR‐BI are shown. The mRNA expression was determined by real‐time quantitative RT‐PCR in placental tissues and normalized to the reference gene YWHAZ. Values represent the fold change (2^−ΔΔCq^) of the mRNA expression in PE compared to controls. Protein expressions were determined by Western blotting using GAPDH as a reference (loading control). Data are shown as mean ± SEM, *n* = 6–14 (control placentae; gray bar) and *n* = 6–11 (PE placentae; orange bar). For comparison of controls with PE, Student's *t*‐test was applied. *p < .05, **p < .01, ***p < .001. Representative immunofluorescence images were obtained after staining for ABCA1 (green), ABCG1 (green), and SR‐BI (red) in placentae from controls and preeclamptic patients. Nuclei were stained with Dapi (blue). Arrows indicate the apical side of the trophoblast, while arrowheads depict the basal side. The scale bar corresponds to 50 μM. Immunofluorescence is shown for a representative image taken from a total of 3 placentae per group. Figure 6A was created with BioRender.com. ABCA1, ATP‐binding cassette (ABC) transporter A1; ABCG1, ATP‐binding cassette (ABC) transporter G1; CHOL, cholesterol; Dapi, 4′,6‐Diamidino‐2‐Phenylindole, Dihydrochloride; GAPDH, glyceraldehyde 3‐phosphate dehydrogenase; SR‐BI, scavenger receptor class B type 1; YWHAZ, tyrosine 3‐monooxygenase/tryptophan 5‐monooxygenase.

In analogy to the trophoblast cell model, we also determined the gene (by qRT‐PCR) and protein (by Western blotting and immunofluorescence) expression of cholesterol transporters in total placental tissue obtained from women with and without PE (Figure 6A). In PE, the placental gene and protein expression of ABCA1 were not significantly different compared to control tissues (Figure 6B–D). In contrast, the gene and protein expression of ABCG1 (Figure 6E–G) and SR‐BI (Figure 6H–J) were significantly increased in PE compared to control placental tissue. This is in full agreement with the results obtained in the trophoblast cell model. Taken together, these results suggest that cholesterol transport activity is altered in PE placentae contributing to disturbed cholesterol homeostasis.

## 4 DISCUSSION

We have investigated several different variables linked to cholesterol homeostasis both in a published cell model, mimicking important features of PE,^38^ and in placental tissue from women who had PE. Comprehensive results from these studies, highlighting different aspects of cholesterol homeostasis, suggest a role for hypoxia in the dysregulation of cholesterol synthesis and cholesterol transport in PE. Although these changes in cholesterol homeostasis may occur only during a short period during pregnancy, they could have long‐term effects on the offspring, including an increased risk of developing CVD. The concept that disturbed lipid homeostasis may contribute to the development or severity of PE is also reflected in the current clinical prophylaxis of PE where statins, metformin, and sulfasalazine are being used and future drugs may include, among others, lipoprotein(a)‐lowering agents.^53^, ^54^, ^55^, ^56^, ^57^

Here, we demonstrate that trophoblast cells exposed to 24 h of hypoxia or H/R exhibit increased intracellular total cholesterol levels. The elevated cholesterol content was mainly due to an increase in non‐esterified (free) cholesterol, similar to what was shown in the macrophage cell line J774 after hypoxia.^37^ Concomitantly, the intracellular accumulation of non‐esterified cholesterol under hypoxic conditions was associated with a significant reduction in the mRNA expression of ACAT1, an enzyme catalyzing the esterification of free cholesterol. These findings suggest that indeed a reduction in the esterification process results in an accumulation of intracellular non‐esterified cholesterol. Taken together, these metabolic adaptations underline that the established in vitro model with primary trophoblast cells—exposed to hypoxia or H/R—reflects several characteristics found in PE. Hence, placentae from preeclamptic pregnancies have been reported to show an accumulation of cholesterol crystals.^12^ This is in line with our current experiments demonstrating an increase in non‐esterified cholesterol in the placentae from women who had PE, compared to controls, using the probe GST‐PFO. Previously, we also demonstrated that in this cell model, the exposure to hypoxia and H/R causes the activation of three main proteins involved in the activation of the inflammasome complex, namely, the NLR family pyrin domain containing 3 (NLRP3), interleukin 1β, and caspase 1.^38^ Although the formation of cholesterol crystals results mainly from cholesteryl esters, in vitro studies in macrophages revealed that this process also occurs intracellularly upon the accumulation of non‐esterified cholesterol.^58^, ^59^, ^60^ Considering these findings in the context of PE, the observed accumulation of non‐esterified cholesterol in placentae from women with PE may lead to the deposition of cholesterol crystals in the placenta following activation of the inflammasome complex.

To understand whether the accumulation of cholesterol is a biological response to increased cellular cholesterol uptake, we measured the mRNA abundance of the LDLR. No difference was observed between controls and trophoblasts exposed to hypoxia or H/R, suggesting that cholesterol uptake was not affected by hypoxia. However, since it has been previously shown that the capacity of LDLR‐mediated cholesterol uptake cannot only be deduced from mRNA and protein levels of the LDLR,^41^ further studies are needed to decisively conclude on this aspect. We then tested the hypothesis that the observed accumulation of intracellular cholesterol in trophoblasts exposed to hypoxia is a consequence of increased cellular cholesterol biosynthesis under these conditions. Thus, we measured the mRNA expression of HMGCR and HMGCS1, the rate‐limiting enzyme for cholesterol biosynthesis and metabolic conversion of acetyl‐CoA. The observed increase in HMGCR suggests that hypoxia affects cellular cholesterol synthesis and contributes to the increased intracellular cholesterol levels detected in the trophoblasts. This is not only in agreement with experiments using HepG2 and J774 cells, where hypoxia increased HMGCR activity and expression^37^, ^61^ but also supports our current findings of increased placental HMGCS1 and HMCGR levels in PE. Overall, these results suggest that placentae synthesize more cholesterol in PE, which accumulates in the tissue, and might harm the offspring during and after pregnancy.

Cholesterol homeostasis and steroid hormone synthesis are linked through a complex regulatory network, involving multiple pathways and molecules. Cholesterol serves as a precursor for the synthesis of various molecules, including steroid hormones, such as progesterone.^62^ For this reason, we investigated if the increased synthesis and accumulation of non‐esterified cholesterol may affect the secretion of steroid hormones important for maintaining pregnancy. Moreover, we speculated that exposure to a hypoxic environment may impair trophoblast functions, which could lead to a reduction in the secretion of progesterone and possibly other hormones necessary during pregnancy. Thus, we analyzed the progesterone concentration in the supernatants of the cells exposed to normoxia, hypoxia, and H/R. However, a continuous increase in progesterone secretion was detected during 24 h without significant differences between the groups. This suggests that progesterone synthesis is neither markedly affected by varying intracellular cholesterol concentrations, nor by hypoxic conditions. However, the extent to which hypoxia affects progesterone levels can vary depending on the severity and duration of hypoxia and is also regulated by individual factors and compensatory mechanisms. Though it has been reported that women with PE may have an imbalance in their hormone levels, including low levels of progesterone,^63^ the effect of hypoxia in this context is currently not clear.

SREBPs are transcription factors that regulate the synthesis and uptake of cholesterol and fatty acids.^49^, ^64^, ^65^ While SREBP‐2 activates the expression of genes such as HMCGR and LDLR, SREBP‐1c controls mainly fatty acid synthesis. SREBP‐1a regulates both uptake and cholesterol biosynthesis and fatty acid synthesis.^49^, ^51^, ^65^, ^66^ After exposing the primary trophoblasts to hypoxia, we observed an increase in the mRNA levels of SREBP‐2, which may explain the upregulation of HMGCR found under hypoxic conditions. SREBP‐1a showed similar results as SREBP‐2, while SREBP‐1c was unaffected by hypoxia. Although these are only preliminary results, these findings may indicate that hypoxia has a more pronounced effect on the cholesterol rather than the fatty acid pathway.

Since we found that hypoxia affected several parameters regulating cholesterol homeostasis, we investigated potential regulatory factors and underlying signaling pathways. Generally, hypoxic cells activate various pathways that affect cell signaling and gene regulation.^67^ One of the best‐described mechanisms by which hypoxic cells adapt to their hostile environment is through the activation of the hypoxia‐inducible transcription factors, HIF‐1 and HIF‐2, which avoid degradation by the proteasome under hypoxic conditions and regulate the expression of a wide range of genes.^68^ Some of the downstream targets are involved in lipid metabolism, and thus, there is an indirect connection between HIFs and cholesterol metabolites.^69^, ^70^ Hence, HIF‐1 and HIF‐2 can regulate SREBPs, increase cholesterol synthesis, and impact lipid transport and storage.^71^ Our results in the trophoblast cell model show an increase in HIF‐2α in hypoxia and H/R and a decrease in HIF‐1α in hypoxia. Though HIF‐1α protein levels are tightly regulated by cellular O_2_ concentrations,^72^ they often do not correlate well with HIF‐1α mRNA levels due to post‐translational regulation.^73^ On the other hand, more HIF‐2α‐positive nuclei in trophoblasts have been reported in PE in the second trimester,^74^ compared with normal pregnancy. These findings suggest that high levels of HIF‐2α in trophoblasts lasting beyond the first trimester may adversely affect pregnancy.

Hypoxia can trigger a surge in the generation of ROS within cells. These ROS can elicit a range of effects on cellular metabolism, potentially leading to the perturbation of cholesterol homeostasis.^75^ Chronic oxidative stress is tightly linked with various diseases, notably CVD, where cholesterol dysregulation plays a pivotal role in the development of atherosclerosis.^76^ Nevertheless, it is worth noting that the intricate molecular mechanisms underlying the pathogenesis of PE, including the impact of oxidative stress and disturbed lipid and cholesterol homeostasis, remain largely unclear. In our study, we investigated ROS production in cells following exposure to hypoxia and H/R and found a significant increase in ROS levels compared to normoxia. Prolonged or recurrent periods of hypoxia, coupled with heightened ROS production, can bear substantial implications for overall health. Hypoxia affects trophoblast cells by inducing cellular stress and activating survival mechanisms through HIF pathways. Prolonged hypoxia may lead to apoptosis, impair trophoblast function, and trigger an inflammatory response.^77^ This can have significant implications for pregnancy outcomes and fetal health. To investigate whether the increased ROS production resulted in enhanced cell death, we also measured cell viability using a MTT‐based assay. We observed that the hypoxic conditions applied in our studies and the concomitantly elevated ROS levels had no impact on cell viability.

To study the underlying signaling pathways, we particularly evaluated the effects of hypoxia on the MAPK pathway since the latter was shown to be involved in the phosphorylation of transcription factors^78^ and in regulating cholesterol efflux^52^; both processes were affected in our cell model. The extracellular‐signal‐regulated kinase (ERK) MAPK pathway has been previously shown to phosphorylate SREBP‐2^78^ and particularly ERK1/2 (also known as p44/p42), two kinases of the MAPK signaling pathway, that have been implicated in HIF activation.^68^, ^79^, ^80^ However, despite a trend toward higher MAPK protein levels at reduced O_2_ levels, no statistically significant differences in the ratio P‐p44/42/total p44/42 protein were detected between normoxia and hypoxic conditions. Whether an increase in the number of samples may result in a statistical significance remains to be elucidated. Alternatively, other cascades of the MAPK pathway may be involved in this process such as JNK1/2/3, which was described to participate in the regulation of ABCA1 in response to hypoxia in macrophages.^81^

Another component of cellular cholesterol handling, which in our studies was found to be affected by hypoxia, was cholesterol export. We evaluated cholesterol efflux both to maternal and fetal sera, which contain several cholesterol acceptors, including the apolipoproteins ApoA‐I and ApoE. Though hypoxia and H/R initially promoted the protein secretion of ApoA‐I at 6 and 12 h, no persistent effects were found at later time points for both apolipoproteins. These results indicate that the increased cholesterol efflux, measured 24 h after exposure to hypoxia, was not due to an exacerbated secretion of cholesterol acceptors, but originated from an induced activity of cholesterol transporters.

In this context, there are three main membrane transport proteins expressed in trophoblasts, which are generally accepted to act as cholesterol efflux transporters: ABCA1, ABCG1, and SR‐BI. It has to be noted that SR‐BI is thought to be involved both in the uptake and efflux of cholesterol.^82^, ^83^ In polarized trophoblasts, these transporters are partially located at different sides of the membrane and do not interact with the same cholesterol acceptors. While ABCA1 is mainly expressed at the apical (i.e., maternal‐facing) side of the membrane and effluxes cholesterol to Apo‐A1, ABCG1 is predominantly located at the basal (fetal‐facing) side promoting cholesterol efflux to HDL. SR‐BI was shown to be located in both parts of the membrane and mediates efflux to HDL.^34^, ^35^, ^41^, ^84^ As expected from the cholesterol efflux results, we indeed found differences in the mRNA and protein expression of the three transport proteins under hypoxic conditions. Our results suggest that the increased cholesterol efflux to maternal serum is mainly mediated by SR‐BI, while the enhanced cholesterol transport to fetal serum is associated with increased activity of both ABCG1 and SR‐BI. These findings are supported by studies demonstrating in macrophages an increased ABCG1‐/SR‐BI‐mediated cholesterol efflux to HDL when the maternal and fetal serum was obtained from women with PE, while ABCA1‐mediated efflux was decreased.^85^ Our data also suggest a diminished activity of ABCA1 under hypoxic conditions, as we found a decreased protein expression of ABCA1 under hypoxia and H/R.

**FIGURE 7:**
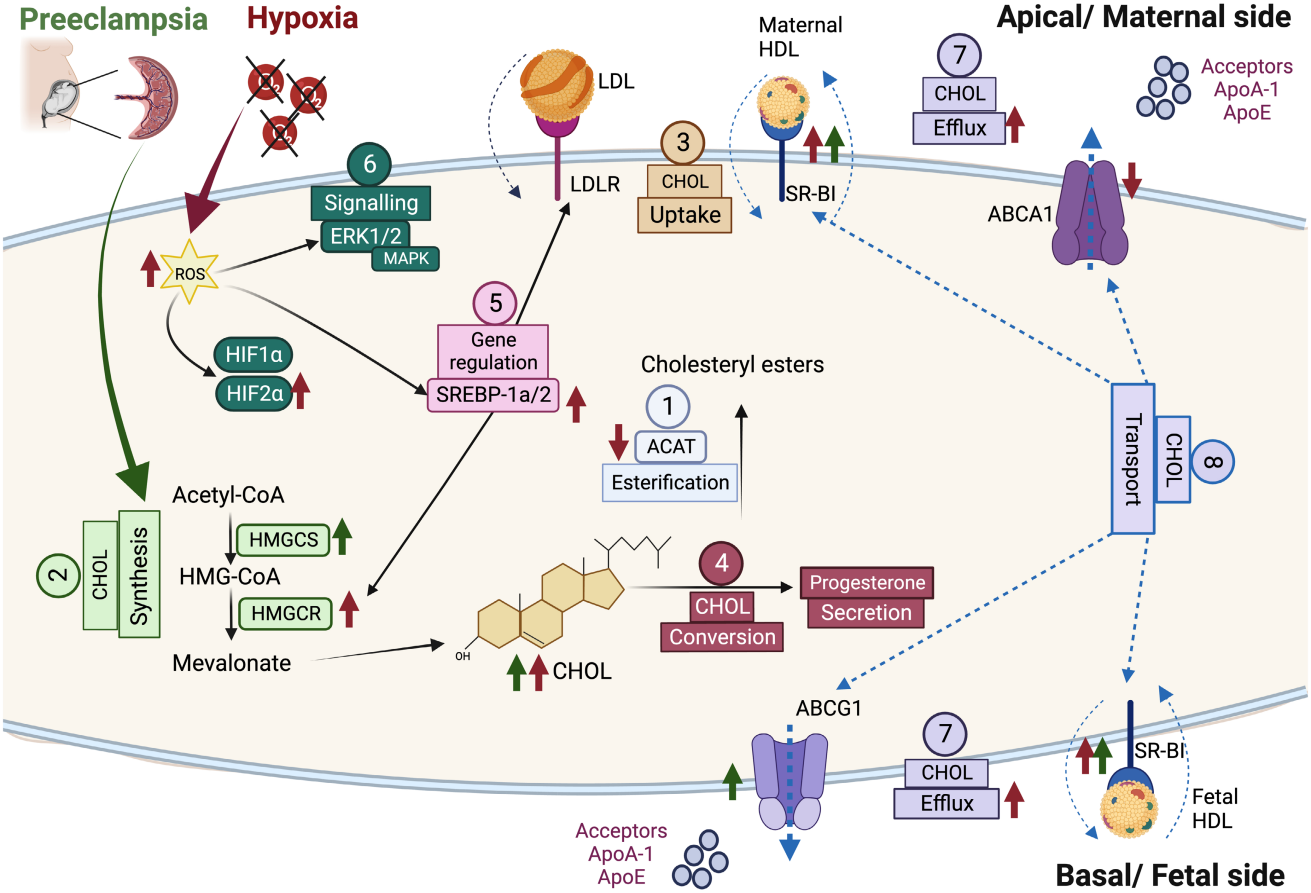
Key aspects of cholesterol homeostasis are altered in a primary trophoblast cell model of preeclampsia and preeclamptic placental tissues. Our findings revealed significant alterations in various parameters regulating cholesterol homeostasis in response to acute hypoxia (as demonstrated in the trophoblast cell model) and under chronic hypoxic conditions as observed in preeclampsia (PE). (1) Intracellular cholesterol content was markedly increased in both the trophoblast cell model and in PE placentae. This increase primarily stemmed from non‐esterified cholesterol, likely resulting from reduced activity of the enzyme ACAT1, leading to a compromised esterification process. (2) Intracellular cholesterol synthesis was augmented under hypoxic conditions in the trophoblast cell model, mirroring the observed trend in PE placentae, ultimately contributing to elevated cholesterol levels within trophoblast cells. (3) Cholesterol uptake mediated by LDLR remained unaffected by hypoxia, though this still has to be confirmed on a functional level. (4) The conversion of cholesterol to progesterone remained unaltered. (5) The expression of SREBP‐1a and SREBP‐2 increased following exposure to hypoxia. (6) Conversely, the signaling of ERK1/2 did not exhibit significant alterations under the hypoxic conditions applied in this study. (7) Cholesterol efflux to both maternal and fetal serum was increased. The significantly enhanced cholesterol efflux is not attributed to an enhanced secretion of cholesterol acceptors but is likely mediated by (8) increased activity of the cholesterol transporters ABCG1 and SR‐BI. Red arrows represent changes induced by hypoxia in the trophoblast cell model, while green arrows correspond to alterations detected in preeclamptic placentae. Numbers and solid black arrows refer to the different pathways that were assessed in this study. Dashed blue lines indicate the direction of cholesterol transport. The figure was created with BioRender.com. ABCA1, ATP‐binding cassette (ABC) transporter A1; ABCG1, ATP‐binding cassette (ABC) transporter G1; ACAT1, acetyl‐CoA acyltransferase 1; ApoA‐I, apolipoprotein A type 1; ApoE, apolipoprotein E; CHOL, cholesterol; HDL, high density lipoprotein; HIF, hypoxia‐inducible factor; HMGCR, 3‐hydroxy‐3‐methylglutaryl‐CoA reductase; HMGCS1, 3‐hydroxy‐3‐methylglutaryl‐CoA synthase 1; LDLR, low density lipoprotein (LDL) receptor; MAPK, mitogen‐activated protein kinases: (ERK1/2: the extracellular‐signal‐regulated kinase); PE, preeclampsia; SR‐BI, scavenger receptor class B type 1; SREBPs, sterol regulatory element binding proteins.

In summary, our data demonstrate that hypoxic conditions affect several pathways of cholesterol homeostasis in primary trophoblast cells. Similar changes could be partially also detected in the placentae of women with PE, indicating that this cell model is a suitable tool for studying cellular processes involved in the pathogenesis of PE. Figure 7 summarizes the main findings: (1) The intracellular total cholesterol content was elevated both in the trophoblast cell model and placentae of women with PE. This increase was mainly attributable to non‐esterified cholesterol and is supposedly due to reduced activity of the enzyme ACAT1 resulting in a diminished esterification process; (2) intracellular cholesterol synthesis was increased by hypoxia in the cell model and in analogy also in the placentae of women with PE, leading to high cholesterol levels in trophoblast cells; (3) cholesterol uptake mediated by LDLR was not altered by hypoxia; (4) the conversion of cholesterol to progesterone was maintained; (5) expression of SREBP‐1a and SREBP‐2 was increased after hypoxia; (6) but the signaling of ERK1/2 was not significantly altered under hypoxic conditions; and (7) cholesterol efflux to both maternal and fetal serum was increased. The increased efflux was not due to an enhanced secretion of cholesterol acceptors but is presumably mediated by (8) increased activity of the cholesterol transporters ABCG1 and SR‐BI.

However, it is worthwhile to note that the results obtained in our studies have to be interpreted with caution since we faced ethical and technical restrictions. Although our results hint at a hypoxia‐mediated dysregulation of cholesterol homeostasis in the primary trophoblast PE cell model, there is currently no direct evidence showing that such alterations in the placenta per se are induced by hypoxia. These data, however, are important to confirm the direct link between hypoxia and altered cholesterol homeostasis in preeclamptic placenta. Alternatively, our attempts to confirm the outcomes in trophoblast cells isolated from preeclamptic placentas—for ensuring the translation of our findings to the preeclamptic condition—were hindered by ethical restrictions in sample collection and limited availability of preeclamptic tissue.

In conclusion, dysregulation of cholesterol homeostasis was observed at several key metabolic points both in the trophoblast cell model and placentae of women with PE. It appears that under hypoxia and in preeclamptic disease, an exacerbated cholesterol synthesis in trophoblast cells cannot be fully compensated by increased cholesterol export, which finally results in an accumulation of (free) cholesterol within the cells. Both the intracellular accumulation of cholesterol and the increased export of cholesterol toward the maternal and fetal circulation may have critical consequences for both the mother and the fetus during pregnancy and may also result in an increased cardiovascular risk later in life.

## AUTHOR CONTRIBUTIONS

Conceptualization, Barbara Fuenzalida, and Christiane Albrecht; methodology, Barbara Fuenzalida, Maria Jose Yañez; data analysis and interpretation of the results, Barbara Fuenzalida and Christiane Albrecht; recruitment of patients and provision of patient data, Christiane Albrecht, Martin Mueller, Hiten D. Mistry and Andrea Leiva; writing—original draft preparation, Barbara Fuenzalida; writing—review and editing, Christiane Albrecht, Hiten D. Mistry, Andrea Leiva; supervision and visualization of the project, Christiane Albrecht; funding acquisition, Christiane Albrecht and Andrea Leiva. All authors have read and agreed to the submitted version of the manuscript.

## FUNDING INFORMATION

This study was supported by the Swiss 3R Competence Centre (3RCC; grant no OC‐2019‐019, CA) and the Swiss National Science Foundation (grant no. 310030_197408; CA). HDM received a British Heart Foundation Basic Science Intermediate Fellowship (FS/15/32/31604). AL and MJY received support from the Fondo Nacional de Desarrollo Científico y Tecnológico FONDECYT (1190250, 11200592, and 1221362).

## DISCLOSURES

The authors declare that there are no conflicts of interest.

## ETHICS STATEMENT

The study was performed according to the Declaration of Helsinki, with the approval of the Ethics Committee of the Canton of Bern (Basec Nr. 2016–00250) and from the Faculty of Medicine of the Pontificia Universidad Católica de Chile (PUC, ID‐180810004). Informed consent was obtained from all subjects involved in the study.

## Supporting information


Figure S1.

## Data Availability

The data and material that support the findings of this study are available upon reasonable request from the corresponding author (christiane.albrecht@unibe.ch).
