# Supplementary material for: Evidence for hypoxia‐induced dysregulated cholesterol homeostasis in preeclampsia: Insights into the mechanisms from human placental cells and tissues
Source: FASEB J. 2024 Jan 24;38(2):e23431. doi: 10.1096/fj.202301708RR (PMC10953329; doi:10.1096/fj.202301708RR)
Supplement: Supplementary file 1 — Figure S1. [file FSB2-38-0-s001.docx]

Evidence for hypoxia-induced dysregulated cholesterol homeostasis in preeclampsia: insights into the mechanisms from human placental cells and tissues

Barbara Fuenzalida^1^, Maria Jose Yañez^2^, Martin Mueller^3^, Hiten D. Mistry^4^, Andrea Leiva^2^ and Christiane Albrecht^1,5^*

^1^ Institute of Biochemistry and Molecular Medicine, Faculty of Medicine, University of Bern, 3012 Bern, Switzerland.

^2^ School of Medical Technology, Faculty of Medicine and Science, Universidad San Sebastián, Santiago 7510157, Chile

^3^ Division of Gynecology and Obstetrics, Lindenhofgruppe, Bern, Switzerland.

^4^ Department of Women and Children´s Health, School of Life Course and Population Health Sciences, King´s College London, London WC2R 2LS, UK.

^5^ Swiss National Center of Competence in Research, NCCR TransCure, University of Bern, 3012 Bern, Switzerland.

**Supplementary Figure 1**

Supplementary Figure 1: Uncropped image for Figure 2I. P-p44/42 MAPK and total-p44/42 MAPK in the hypoxia and H/R cell model.

**Supplementary Figure 2**

Supplementary Figure 2: Uncropped images for Figure 4C. ABCA1 and beta-actin in the hypoxia and H/R cell model.

**Supplementary Figure 3**

Supplementary Figure 3: Uncropped images for Figure 4E. ABCG1 and beta-actin in the hypoxia and H/R cell model.

**Supplementary Figure 4**

Supplementary Figure 4: Uncropped images for Figure 4G. SR-BI and beta-actin in the hypoxia and H/R cell model.

**Supplementary Figure 5**

Supplementary Figure 5: Uncropped images for Figure 5F. HMGCS and GAPDH in preeclamptic placental tissues.

**Supplementary Figure 6**

Supplementary Figure 6: Uncropped images for Figure 5G. HMGCR and GAPDH in preeclamptic placental tissues.

**Supplementary Figure 7**

Supplementary Figure 7: uncropped images for Figure 6C. ABCA1 and GAPDH in preeclamptic placental tissues.

**Supplementary Figure 8**

Supplementary Figure 8: uncropped images for Figure 6F. ABCG1 and GAPDH in preeclamptic placental tissues.

**Supplementary Figure 9**

Supplementary Figure 9: uncropped images for Figure 6I. SR-BI and GAPDH in preeclamptic placental tissues.

**Supplementary Figure 10**

Supplementary Figure 10: negative control for immunofluorescence in preeclamptic placental tissues shown in Figures 5B, 6D, 6G and 6J. The secondary antibodies were Alexa Fluor 488-conjugated goat anti-rabbit, Alexa Fluor 568-conjugated goat anti-mouse IgG and Alexa Fluor 594-conjugated goat anti-goat.
